# Duodenum preserving pancreatectomy in chronic pancreatitis: Design of a randomized controlled trial comparing two surgical techniques [ISRCTN50638764]

**DOI:** 10.1186/1745-6215-7-12

**Published:** 2006-05-08

**Authors:** Jörg Köninger, Christoph M Seiler, Moritz N Wente, Margot A Reidel, Emre Gazyakan, Ulrich Mansmann, Michael W Müller, Helmut Friess, Markus W Büchler

**Affiliations:** 1 Department of General Surgery, University of Heidelberg, Germany; 2 Department of General Surgery, Center for Clinical Studies, University of Heidelberg, Germany; 3 Institute for Medical Biometrics and Informatics, University of Heidelberg, Germany

## Abstract

**Background:**

Chronic pancreatitis is an inflammatory disease which is characterized by an irreversible conversion of pancreatic parenchyma to fibrous tissue. Beside obstructive jaundice and pseudocyst formation, about half of the patients need surgical intervention due to untreatable chronic pain during the course of the disease. In most of the patients with chronic pancreatitis, the head of the pancreas is the trigger of the chronic inflammatory process. Therefore, resection of pancreatic head tissue must be the central part of any surgical intervention. However, it is unclear to which extent the surgical procedure must be radical in order to obtain a favourable outcome for the patients.

**Design:**

A single centre randomized controlled, superiority trial to compare two techniques of duodenum preserving pancreatic head resection. Sample size: 65 patients will be included and randomized intraoperatively. Eligibility criteria: All patients with chronic pancreatitis and indication for surgical resection and signed informed consent. Cumulative primary endpoint (hierarchical model): duration of surgical procedure, quality of life after one year, duration of intensive care unit stay, duration of hospital stay. Reference treatment: Resection of the pancreatic head with dissection of the pancreas from the portal vein and transsection of the gland (Beger procedure). Intervention: Partial Resection of the pancreatic head without transsection of the organ and visualization of the portal vein (Berne procedure).

Duration: September 2003-October 2007.

**Organisation/responsibility:**

The trial is conducted in compliance with the protocol and in accordance with the moral, ethical, regulatory and scientific principles governing clinical research as set out in the Declaration of Helsinki (1989) and the Good Clinical Practice guideline (GCP). The Center for Clinical Studies of the Department of Surgery Heidelberg is responsible for planning, conducting and final analysis of the trial.

## Background

### Medical problem

Chronic pancreatitis is an inflammatory disease, which is characterized by progressive and irreversible destruction of pancreatic parenchyma and transformation into fibrous tissue. The leading symptom of chronic pancreatitis is chronic pain, which requires surgical intervention in about 50% of the patients [1].

Despite of the fact that the etiopathogenesis of pain in chronic pancreatitis is not fully understood, the concept of a neuroimmune interaction in the head of the pancreas is in accordance with the observation that simple drainage procedures seldom lead to satisfactory pain relief in the long run in patients with an enlarged pancreatic head [2-6]. Only surgical techniques that include resection of the inflammatory mass in the pancreatic head have shown to be successful in the treatment of this disease [7,8].

### The Beger procedure (reference treatment)

**Figure 1:**
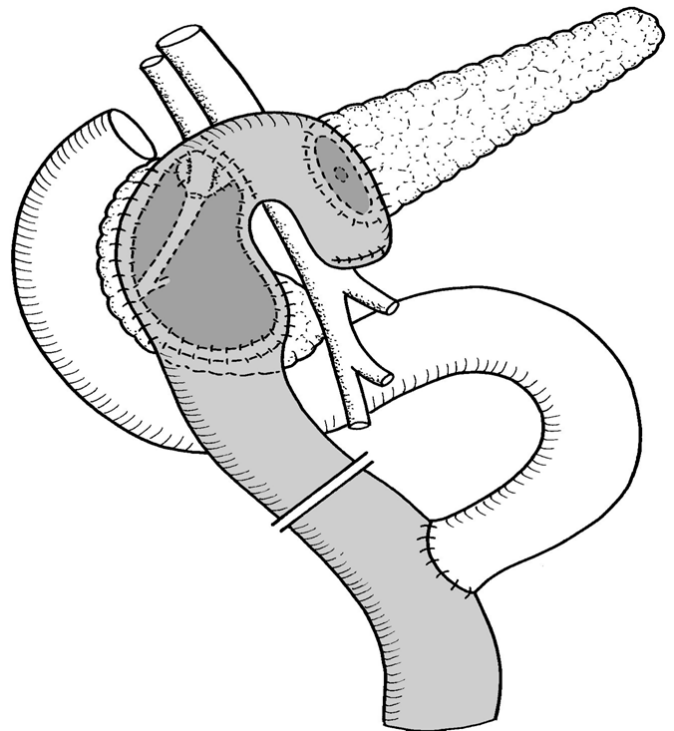
**Beger procedure (reference treatment)**. Reconstruction after duodenum preserving pancreatic head resection with a Roux en-Y jejunal loop as side-to-end and side-to-side pancreatico-jejunostomy.

In 1972, H.G. Beger for the first time described the technique of duodenum preserving pancreatectomy in the treatment of patients with chronic pancreatitis (Fig. 1) [9,10]. The rationale of this intervention is the resection of the inflammatory mass in the pancreatic head without large loss of unaffected parenchyma and maintenance of the duodenal passage. Different studies have shown that this organ preserving technique is at least as effective as the more radical Kausch-Whipple procedure (pancreatico-duodenectomy) regarding pain control (effective pain diminishing in >80% within 5 years), but postoperative morbidity and the incidence of diabetes mellitus are lower [1,11-13].

### The Berne procedure (selection of intervention)

Over the years, different variations of the duodenum preserving technique have been introduced. In 1985, Frey and Smith described a modification in which a longitudinal pancreatico-jejunostomy is combined with a local resection of the pancreatic head [14,15]. This technique combines the principle of duodenum preserving pancreatic head resection with the drainage of the main pancreatic duct. In 1998, similar to the Frey technique, Izbicki et al. combined the duodenum preserving resection of the pancreatic head with a V-shaped incision of the body of the pancreas in order to also reach II° and III° pancreatic side branches. The results obtained with this technique are very similar to the original Beger technique. Thirty patients were operated without mortality. After a median follow up of 30 months, 92% of the patients were pain free with preserved endocrine and exocrine function [16].

The main advantage of these variations of the original Beger procedure lies in the fact that the dissection of the pancreas from the portal vein can be avoided, which is regarded as the most hazardous part of the intervention [4]. Especially in case of portal vein thrombosis, the division of the pancreatic body from the portal vein, which is an essential step in the Beger (and also Kausch-Whipple) procedure, can be very demanding. Bleeding from the portal vein in this situation can be extremely difficult to control.

**Figure 2:**
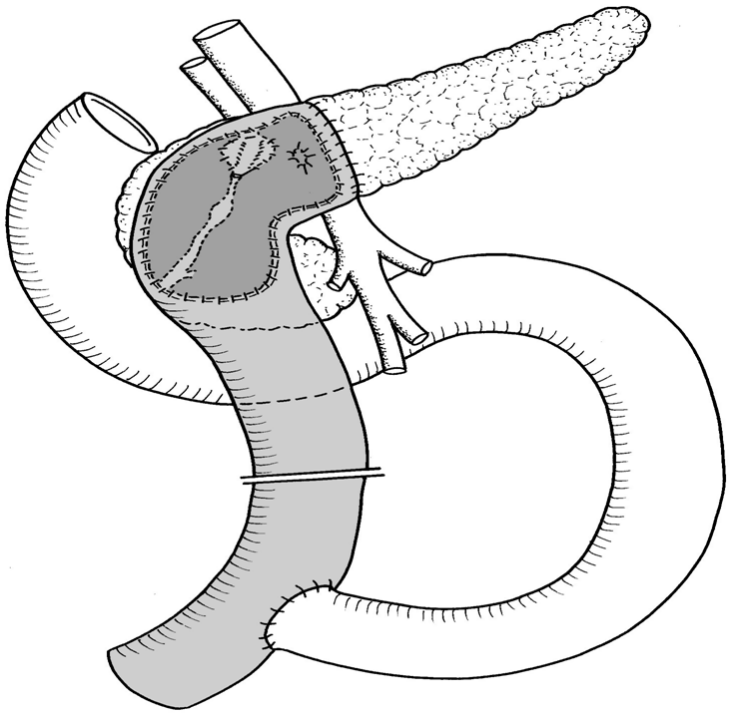
**Berne procedure (intervention)**. Reconstruction after duodenum preserving pancreatic head resection with a Roux en-Y jejunal loop as side-to-side pancreatico-jejunostomy.

To combine the advantages of the original Beger procedure with the Frey technique, we developed an additional modification which consists in a duodenum preserving resection of the pancreatic head in analogy to the original Beger technique, with the difference that a small shape of pancreatic tissue remains on the anterior wall of the portal vein in a way that the hazardous division of vein and pancreatic body is avoided (Fig. 2) [17].

## Study design

### Aim of the study

The objective of this trial is to compare two different surgical techniques for the treatment of chronic pancreatitis with regard to complication rates, length of operation, length of intensive care treatment, length of hospital stay, exocrine/endocrine pancreatic function and quality of life.

### Number of patients needed

As estimated effect size a difference of mean operating time of one hour between the two groups and a variability of this difference in between the expected absolute effect (standard deviation of one hour) is defined. Groups of 22 patients each would permit the verification of the null-hypothesis with an α-error set at 0.05 and a β-error at 0.10, yielding a power of 90%.

To evaluate the true efficacy of the Berne procedure a per protocol analysis is necessary. For the external validity of the study an intention to treat approach for analysis is requested. Due to the nature of complex surgery such as a pancreatic resection it can be expected that one third of all randomised patients will not receive the intended procedure. Therefore at least 65 patients should be randomised.

## Eligibility criteria

### Inclusion criteria

• Patients of any age (>18 ys)

• Expected survival time more than 24 months

• Informed consent

### Exclusion criteria

• Participation in another intervention trial that would interfere with the intervention and outcome of this study

• Severe psychiatric disorders or neurological diseases

• Lack of compliance

• Drug and/or alcohol abuse according to local standards

### Ethics, study registration and consent

The final protocol was approved by the independent ethics committee of the University of Heidelberg. The study was registered at Current Clinical Trials (ISRCTN No. 50638764). Patients who are scheduled for duodenum preserving pancreatic head resection had a pre-treatment visit to give the informed consent. During this visit the patient will be screened and informed about the trial. In the course of the conversation, the study procedure, risks, benefits and data management will be clarified in detail.

## Randomisation and procedures for minimising bias

### Minimising systematic bias

In order to achieve comparable groups for known and unknown risk factors, randomization will be performed. The random allocation sequence was generated by a free available randomization software tool [18] by a trained independent study nurse not further involved in this trial. She also prepared the sealed and opaque envelopes for the randomisation procedure in the operation theatre. The sealed randomization list was stored in the investigator file. A sufficient number of patients will be recruited according to the sample size calculation in order to prevent random error. Patients will get randomised intra-operatively by an independent study nurse or anaesthesiologist once the surgeon decides that clinical equipoise is given, usually after the pancreas has been prepared and mobilised (Kocher's maneuver). All interventions will be done as described below. Patients in whom the randomised procedure cannot be performed due to technical or anatomical reasons will be analysed according the intention to treat principle.

### Minimising treatment bias

The participating surgeons are all experienced in pancreatic surgery. Annually more than 300 resections of the pancreas are performed in Heidelberg. Both procedures used in this trial are supervised by two authors (H.F., M.W.B.) in order to guarantee correct execution according to the protocol (intraoperative surgical monitoring). Concomitant treatment will be equal for both groups including antibiotic prophylaxis, use of sandostatin and anaesthesia.

### Minimising measurement bias

An independent study nurse will document and monitor the procedure in the operating theatre. Blinding in this phase of the trial is not possible due to the research question.

Standardized patient interviews will be performed by blinded study nurses 6, 12 and 24 months after surgery, using EORTC QLQ-C30 questionnaire and the disease specific module for pancreatic diseases EORTC QLQ-PAN26 [19,20].

## Study treatment

After a midline laparotomy, wide exposure of the pancreas is obtained by opening of the gastro-colic ligament. The duodenum and the pancreatic head are then mobilized by an extended Kocher maneuver. If there are any doubts on the dignity of the pancreatic head tumour, biopsies will be taken and pathological examination will be performed.

After exhibition of the pancreas, the randomisation will be performed using sequentially numbered, opaque, sealed envelopes; according to the allocation, the procedure will be continued.

### Beger procedure (reference treatment)

The pancreas is gently lifted and subtle dissection is performed between the mesenteric vein and the body of the pancreas, taking care to avoid injury to the major retropancreatic vessels and smaller side branches. After this step, multiple stay sutures (4/0 PDS) are placed around the parenchyma of the pancreatic head to assign resection margins. This is important to avoid inadvertent injury to the duodenum and assures that a lamella of pancreatic parenchyma along the loop of the duodenum remains and assures blood supply. The subtotal resection of the pancreatic head starts with the transsection of the pancreas above the portal vein proceeding towards the prepapillary common bile duct. In all phases of the intervention meticulous haemostasis is required. Reconstruction is performed with a Roux-en-Y loop of the jejunum with an end-to-side pancreatico-jejunal anastomosis and another side-to-side anastomosis of the jejunal loop with the remaining hollowed pancreatic head.

### Berne procedure (intervention treatment)

Both the dissection of the pancreatic body from the portal vein and the transsection of the pancreas over the portal vein is not done with the consequence that only one cavity results. Also in this case, multiple stay sutures mark the dissection margin and support blood sparing surgery. After subtotal resection of the pancreatic head, reconstruction is performed in analogy to the original Beger-technique with a Roux-en-Y jejunal loop and side-to-side pancreatico-jejunostomy.

Independently of the type of resection (Beger or Berne procedure), in case of stenosis of the intrapancreatic segment of the common bile duct which cannot be decompressed by resecting the circumferential fibrous tissue or in case of accidental opening of the common bile duct during resection, an additional bilio-digestive anastomosis will be performed.

## Primary and secondary endpoints

### Primary endpoints

The primary endpoint is combined out of four components. In order to adjust for multiple testing a hierarchical model is used.

1. Duration of surgical procedure [min]

2. Quality of life (EORTC QLQ-C30 questionnaire and the disease specific module for pancreatic diseases EORTC QLQ-PAN26) at 12 months after the intervention

3. Duration of stay on the intensive care unit

4. Duration of postoperative hospital stay

### Secondary endpoints

Frequencies of early and late onset complications such as intra- or postoperative bleeding with subsequent need for blood transfusion, pancreatic fistula, postoperative pulmonary complications, wound infections and re-laparotomy; exocrine and endocrine pancreatic function as determined by levels of HbA1c and stool elastase.

## Adverse events and serious adverse events

The term *adverse event *(AE) covers any sign, symptom, syndrome, or illness that appears or worsens in a patient during the period of observation in the clinical trial and that may impair the well-being of the patient. The term also covers laboratory findings or results of other diagnostic procedures that are considered to be clinically relevant. A *serious adverse event (SAE) *is any adverse event that occurs at any time during the period of observation that results in death, is immediately life-threatening, requires or prolongs hospitalization, or results in persistent or significant disability or incapacity.

AEs will be reported to the principle investigator in regular intervals throughout the study.

SAEs which are meet one of definitions of the secondary endpoints are treated as SAEs regarding to documentation, but have not to be reported to the sponsor/principle investigator within 24 h.

## Analysis

A flow chart according to the CONSORT statement is included [21]. The baseline characteristics of patients in both groups will be given in a table.

Comparisons will be made of the primary endpoints of both intervention groups for all patients included in the study on an intention to treat basis. Furthermore per protocol analysis will be performed as well as including only patients strictly treated according to the study protocol.

**Figure 3:**
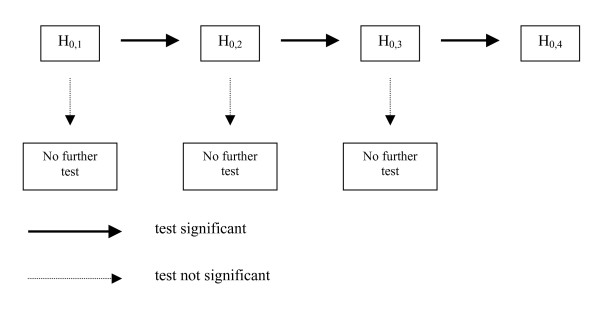
**Flow chart**. If the null hypothesis of no difference can be rejected the other components can be tested in a hierarchical way.

The outcome measures of the primary endpoint will be tested for significance with the Mann-Whitney Test (The test of H_0,1 _no *difference of duration of the surgical procedure *on the level of 5%). If the null hypothesis of no difference can be rejected the other components can be tested in a hierarchical way as follows (Fig 3):

1. The test of H_0,2 _*Quality of life in both groups is not equivalent *is an equivalence test. Based on preliminary experiences with the EORTC QLQ-C30/PAN26 questionnaires in pancreatic cancer patients [20], we estimated baseline raw score values in the range of about 60 (SD 25) before therapy. When transformed into a percentage scale (with 100% indicating perfect health) this represents values of 45% (SD 18%). After surgery, quality of life can be estimated to improve up to value of 75% (SD 18%). We assume equivalence for the two resection techniques, if the two-sided 95% confidence intervals for difference in quality of life fall within the interval of +/-15%. The value of 15 as delta was chosen, because the overall effect of surgery is in the range of about 30 percentage points. Therefore, the minimal clinically relevant difference can be estimated by halving this effect of surgery. To show equivalence for a delta of 15 (SD 18) with alpha = 5% and beta = 20%, a sample size of 60 patients is required [22].

2. The test of H_0,3 _*Length of stay on the intensive care unit of both groups is not equivalent *is an equivalent test on the level of 5%. The zero-hypothesis is declined if the 90% confidence interval for the difference of mean values of ICU stay in hours for both groups is in between [-12/12 hours].

3. The test of H_0,4 _*Hospital stay on both groups is not equivalent *is an equivalent test on the level of 5%. The null-hypothesis is declined if the 90% confidence interval for the difference of mean hospital stay in days for both groups is in between [-2/2 days].

Once the null hypothesis cannot be rejected no further testing is applied. All secondary endpoints will be analyzed using descriptive and graphical methods.

Safety-related data will be analyzed with respect to frequency of:

• Severe bleeding intra- or postoperatively requiring blood transfusion

• Laparotomy

• Pancreatic fistula

• Pulmonary infection and wound infection are secondary endpoints, but are also defined as adverse events. Severe intraoperative bleeding with need for blood transfusion will even always be considered a serious adverse event.

Safety-related data will be analyzed with respect to frequency of:

• Serious adverse events and adverse events stratified according to the organ-systems

• Adverse events stratified by severity

• Adverse events stratified by causality

## Study organisation

All patients scheduled for duodenum preserving pancreatic head resection at the Department of Surgery, University of Heidelberg, will be referred to the Center for Clinical Studies in Surgery (KSC) [23] and screened by members of the KSC. Approximately 80 patients per year undergo a duodenum preserving pancreatic head resection for chronic pancreatitis at the Department of Surgery, University of Heidelberg. The estimated time frame to randomize 65 patients is approximately 18 months.

Data will be recorded in clinical reporting files. Double data entry will be done by two independent staff members and all analysis are performed with SPSS (SPSS, Chicago, IL) after plausibility controls and closure of the database.

## Competing interests

The author(s) declare that they have no competing interests.

## Authors' contributions

J.K. and C.M.S participated in the design and coordination of the study and drafted the manuscript. M.N.W., M.E.R., E.G., and M.W.M. participated in the design and coordination and helped to draft the manuscript. U.M. participated in the statistical design and helped to draft the manuscript. H.F. and M.W.B. conceived the study and supervised the coordination of the study. All authors read and approved the final version of the manuscript.
